# The role of nanocomposites against biofilm infections in humans

**DOI:** 10.3389/fcimb.2023.1104615

**Published:** 2023-02-28

**Authors:** Anand Varma, Ashish Warghane, Neena K. Dhiman, Neha Paserkar, Vijay Upadhye, Anupama Modi, Rashmi Saini

**Affiliations:** ^1^ Arundeep Akshay Urja Pvt. Ltd. Gorakhpur, Uttar Pradesh, India; ^2^ School of Applied Sciences and Technology (SAST), Gujarat Technological University, Ahmedabad, Gujarat, India; ^3^ Department of Zoology, Gargi College, University of Delhi, Delhi, India; ^4^ Faculty of Life Sciences, Mandsaur University, Mandsaur, Madhya Pradesh, India; ^5^ Centre of Research for Development (CR4D), Parul University, Vadodara, Gujarat, India

**Keywords:** nanocomposite, biofilm, human disease, bacteria, infections

## Abstract

The use of nanomaterials in several fields of science has undergone a revolution in the last few decades. It has been reported by the National Institutes of Health (NIH) that 65% and 80% of infections are accountable for at least 65% of human bacterial infections. One of their important applications in healthcare is the use of nanoparticles (NPs) to eradicate free-floating bacteria and those that form biofilms. A nanocomposite (NC) is a multiphase stable fabric with one or three dimensions that are much smaller than 100 nm, or systems with nanoscale repeat distances between the unique phases that make up the material. Using NC materials to get rid of germs is a more sophisticated and effective technique to destroy bacterial biofilms. These biofilms are refractory to standard antibiotics, mainly to chronic infections and non-healing wounds. Materials like graphene and chitosan can be utilized to make several forms of NCs, in addition to different metal oxides. The ability of NCs to address the issue of bacterial resistance is its main advantage over antibiotics. This review highlights the synthesis, characterization, and mechanism through which NCs disrupt Gram-positive and Gram-negative bacterial biofilms, and their relative benefits and drawbacks. There is an urgent need to develop materials like NCs with a larger spectrum of action due to the rising prevalence of human bacterial diseases that are multidrug-resistant and form biofilms.

## 1 Introduction

The extensive association of the encapsulated immobile microorganisms that cling to the solid surface with the aid of a self-generated hydrated extracellular matrix is called biofilm. These biofilms are often found on damp surfaces forming a slippery covering. The environment is not always favourable outside the bacterial cell, such as in the biological system. There is always a chance that the bacteria would encounter host immune cells (phagocytes) and their product in the form of antibodies. In the non-biological system, bacteria get exposed to hostile environments such as heat, cold, and UV light. This matrix is composed of extracellular polymeric substances such as carbohydrates and exopolysaccharides (Prosser et al., 1987; Lawrence et al., 1991), which accompanies the adhering of the giant bacterial framework together, thereby providing strength and protection from these extreme biological and non-biological factors. The microscopic examination of the biofilm is tedious because dehydration causes the matrix to collapse. This biofilm comprises 82%–85% of the extracellular matrix and 15%–20% of the microbial consortium (Cowan 2011). Initially, Antony van Leeuwenhoek investigated the biofilm from his dental plaque and observed that sessile bacteria contribute to biofilm formation. Later, it was found that the aggregation pattern of the bacteria depends on the different bacterial species. One bacterial species can form a network with other bacterial species (Costerton et al., 1999). Fundamentally, through Brownian motion, the planktonic bacteria encounter the exposed surface, where they are subjected to the abhorrent electrostatic force. This immotile mass of cells is nutritionally deficient; hence, some of these sessile cells skip the matrix and become motile to access the nutrients readily and reproduce expeditiously. The cells in the motile phase are also called plankton.

It is now understood that approximately 40%–80% of bacterial cells on earth can form biofilms (Flemming and Wuertz, 2019). Bacterial biofilms are embedded communities of bacteria apprehended together by a self-produced polymer matrix mainly consisting of polysaccharide proteins and extracellular nucleic acid. In the human body, biofilm is found over the skin, mucosa, and teeth. The best-known example of biofilm is plaque. It is shown that biofilms are found in implantable medical devices. Devices that partly penetrate the skin, such as a central venous catheter, can become coated with bacterial biofilms. It is found on fully implanted devices such as artificial hip or knee joints. The biofilm of *Escherichia coli* is frequently found on urinary catheters. The studies also revealed that biofilm forms inside the patients having chronic lung infection, cystic fibrosis, and lung damage (Kostakioti et al., 2013). There are various biofilm-creating pathogens that are related to chronic upper respiratory infections (*Pseudomonas aeruginosa*) (Koch and Hoiby, 1993; Govan and Deretic, 1996), urinary tract infections (UTIs) (uropathogenic *E. coli* [UPEC], *Klebsiella pneumoniae*), periodontitis (mixed biofilms of *Streptococcus mutans* and other bacteria), and catheter-induced and other device-associated infections (*E. coli, Enterococcus faecalis*, and others) (Venditti et al., 1993; Ferrieres et al., 2007; Jacobsen et al., 2008; Kuramitsu and Wang, 2011). The strains of *S. epidermidis* and *Streptococcus aureus* adhere to the ocular infections (Hou et al., 2012).

## 2 Biofilm-related infections

The skin is the largest organ in the human body and is populated by distinct microbes like viruses, bacteria, and fungi (Byrd et al., 2018). The distribution of these distinct microbes depends on not only the skin microhabitat and texture (scorched, damp, and oily) but also the microenvironment of the skin (acidic or nutritionally deficient environment).

**Figure 1:**
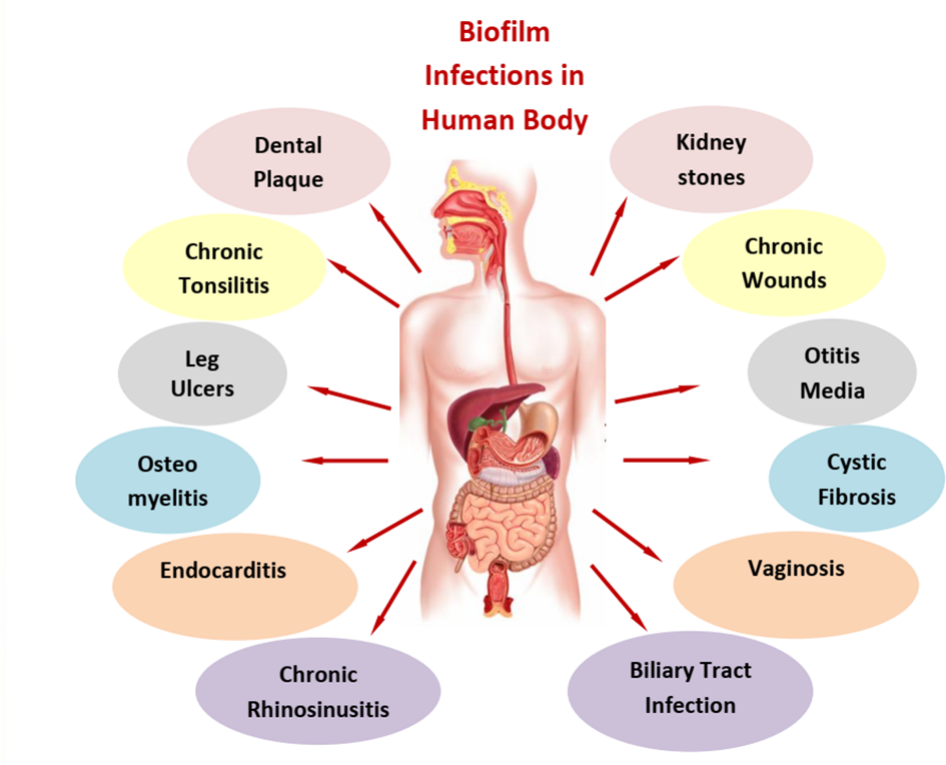
Diagrammatic representation of the biofilm infections in various types of human diseases.

As illustrated in **Figure 1**, biofilms are associated with various human diseases. The primary concern is the face, chest, and abdomen. Dental plaque, otitis media, and chronic rhinosinusitis and chronic tonsillitis are the predominant diseases in the face and neck region of the human body. Dental plaques are seen after the aging of the disease as biofilm was established well enough between teeth and gums. Dental plaque is a biofilm that is both physically and functionally structured. The plaque has a complex microbial makeup that, in good health, builds in an organized manner and is largely stable over time (microbial homeostasis) (Marsh, 2006). Next to this, chronic tonsillitis in the mouth lies near the junction of the mouth and neck. This disease is mainly found in growing children aged between 5 and 15 years. It is primarily due to *Streptococcus* bacteria; a repository of infection in wet and warm folds of the tonsils is a major source for its reoccurrence (Bakar et al., 2018). Otitis media is a middle ear infection that occurs as a result of cold, sore throat, or respiratory infection (Niedzielski et al., 2021), while chronic rhinosinusitis is associated with nasal congestion, mucus discharge from the nose, facial pain, and pressure along with a decreased sense of smell (Karunasagar et al., 2018) and is common in both adults and children. For this, the first line of treatment is nasal sprays. Moving towards the chest, cystic fibrosis and endocarditis are the dominant bacterial diseases. Endocarditis is an infection of the heart’s inner lining (endocardium), usually involving the heart valves. It occurs when germs from elsewhere in the body travel through the blood and attach to the damaged areas of the heart. People with damaged or artificial heart valves or other heart conditions are mostly at risk. *Streptococci* and *Staphylococci* are the main pathogens for such infections (Lerche et al., 2021; Martin et al., 2021). In cystic fibrosis, a lung infection caused by *P. aeruginosa*, great tolerance to antibiotics and resistance to phagocytosis were reported (Høiby et al., 2010). These infections often last for decades. Anti-inflammatory medications along with mucus-thinning drugs can enhance the power of antibiotics in such cases. Finally, in the abdomen area, we have a major concern with biliary tract infection, kidney stones, and vaginosis. In the case of biliary tract infection and kidney stones, insoluble solids of large dimensions are formed that obstruct the passage and disrupt normal functioning. The pathogenesis of brown pigment gallstones, the blockage of biliary stents, and biliary parasitic infestations are related to the formation of bacterial biofilms. *E. coli* and other enteric organisms attach themselves to the surface of an ulcerated biliary mucosa or implanted biomaterials to form a bacterial biofilm. Crystals of calcium bilirubin and other biliary sediments accumulate around the bacterial microcolonies and glycoproteins, resulting in thick and often calcified biofilms obstructing the normal bile flow (Sung et al., 1992). Urinary tract stones are induced by infection with urease-positive pathogens and affect dialysis systems, including peritoneal and central venous catheters (Soto, 2014). In the lower abdomen, vaginal biofilms have the maximum number of cases due to the presence of biofilm-protecting bacteria from antibiotic treatment and serve as a reservoir for the regrowth of pathogens. Vaginal biofilms play a key role not only in bacterial vaginosis pathogenesis but also in its treatment failure and recurrence (Machado et al., 2015). *Lactobacillus* species is one of the major causes of this disease. In osteomyelitis, non-organ-related biofilm formation is clearly evident in bones. In this condition, inflammation of bones in the legs, arm, or spine is observed. Infection in this case can reach bones by traveling through the bloodstream or spreading from nearby tissue or due to injury. Smokers and diabetic individuals are more prone to it. *Staphylococcus* is a major cause of such infection. Artificial joints like hip replacement, metal implants such as screws, and bedsores are other major factors contributing to osteomyelitis.

## 3 Chronic wounds and biofilms

Wounds that last for more than 45 days lead to the formation of chronic wounds, which is an impending clinical problem. These wounds are unable to heal because of disordered physiological processes and wound environments drastically affected by many microbes. These polymicrobial associations involve the arduous interaction between the organisms leading to the pathogenesis of bacterial biofilm-associated infections (Verbrugghe et al., 2021). Several types of communications occur between the microbes associated with biofilms. This association can be cooperative, competitive, or antagonistic (Nadell et al., 2016). There are certain chemicals released by both Gram-positive and Gram-negative bacteria, making it possible for the bacteria to communicate with each other, such as Gram-negative bacteria releasing homoserine lactones or quinolones. Similarly Gram-positive bacteria release short peptides (Sturme et al., 2002). Bacterial biofilms not only offer resistance against antibiotics but also offer resistance against ultraviolet light and heavy metals. Micronutrients and wound exudates, which contribute to the optimal condition of the wound, are breeding hubs for multiple microbes forming biofilms. Approximately 60% of chronic wounds contain biofilms (Omar et al., 2017). As per reports, infection persistence and polymicrobial association are the leading factors for frequent infection. Generally, chronic wounds are primarily classified into three classes, i.e., pressure ulcers, venous leg ulcers (VLUs), and diabetic foot ulcers (DFUs), whereas the secondary class of chronic wound consists of cancer ulcers, inflammatory ulcers, and ischemic ulcers. Microbial profiling of this wound reveals the prevalence of *Serratia*, *Pseudomonas*, *Staphylococcus*, and *Enterobacter*.

### 3.1 Pressure ulcer

Decubitus ulcers or bedsores notoriously known as pressure ulcers have not been studied immensely. Elderly people who shifted to long-term care facilities (LTCFs) are more prone to this type of ulcer, which is commonly seen in medical conditions. These ulcers are formed in bed-rest patients, by the pressure generated on the skin because of friction and the body weight for a longer period. Such initial pressure for approximately 1–2 h leads to pressure-induced cell deformation causing cell death. Pressure ulcers if untreated for a long time can also infect soft tissues, causing osteomyelitis and may even lead to death (in 39.7% of patients) (Rennert et al., 2009). *P. aeruginosa*, *S. aureus*, coagulase-negative *Staphylococcus* (CoNS), and *E. faecalis* are prominent microbes associated with pressure ulcers (Suleman and Percival, 2015). According to the National Pressure Ulcer Advisory Panel (NPUAP) and the European Pressure Ulcer Advisory Panel (EPUAP), pressure ulcers are categorized into four stages of wound infection, i.e., stage I (non-blanchable erythema), stage II (partial thickness), stage III (full thickness skin loss), and stage IV (full thickness tissue loss) (Panel, 2014). Medical sectors are under huge economic strain because of the increased cost of treatment of pressure ulcers, i.e., approximately £1,214 (category 1) to £14,108 (category IV) (Dealey et al., 2012). The peptide nucleic acid-based fluorescence *in situ* hybridization (PNA-FISH) studies by Kirketerp-Møller and colleagues on pressure ulcers revealed the existence of biofilms in it. Polymicrobial biofilm in association with *S. aureus* and *C. albicans* in this ulcer has been responsible for resistance to vancomycin (Harriott and Noverr, 2009).

### 3.2 Venous leg ulcer

Fazli and his colleagues used PNA-FISH to figure out that the VLU had biofilm (Fazli et al., 2011). *P. aeruginosa* is the persistent and prime bacterium responsible for chronic VLU. A leg ulcer is a long-lasting sore that usually develops on the inside of the leg, between the knee and the ankle. Its symptoms include pain, itching, swelling, and discoloration. The hardened skin around the ulcer and the sore may produce a discharge with a foul smell. There are two major causes for its development. It develops after a minor injury due to persistent high pressure in the veins of the legs. When there is high pressure in the veins of the lower leg, the veins become scarred and blocked, resulting in blood pooling in the legs. This is called venous insufficiency. The increased pressure and fluid buildup prevent nutrients and oxygen from reaching tissues. The lack of nutrients causes damage to the tissue and wound formation. Improvement of blood flow in the legs is a temporary method of treatment, and this internal environment facilitates the pathogens to develop a sustained colony as biofilm.

### 3.3 Diabetic foot ulcer

Approximately 15%–25% of the patients suffering from diabetes mellitus are prone to developing DFU (Armstrong et al., 2017). Diabetic foot complications are the most common cause of non-traumatic lower extremity amputations. A round, red crater in the skin bordered by thickened callused skin is the identifying feature of this disease. Poor control of blood sugar, foot deformities, bad foot care, shoes that do not fit right, peripheral neuropathy, poor circulation, and dry skin are the root causes. Slow recovery is one of the biggest problems with getting rid of this disease. This makes it easier for bacteria to take over the affected area, which causes biofilm to form. Routine practice for treatment includes optimizing diabetes control to reduce neuropathic and vascular complications. Implementing MADADORE (M = metabolic/medication; A = assessment; D = debridement; A = antibiotics; D = dressing; O = offloading; R = referral; E = education) is now being talked about in the medical community as a way to control it (Lazzarini et al., 2019).

## 4 Therapeutic strategies against bacterial biofilms

Several opportunistic pathogens evoke skin infection by the formation of biofilm, which is a crucial virulence factor. Traditional therapies are inefficient for treating the obstinate factor of biofilm and antibiotic tolerance. Biofilm forms the protective covering around the bacteria that prevents the intake of antibacterial therapies. Nanoparticle-based curative therapies proved to be pivotal in counteracting these robust biofilms, thereby making the entry of the drugs feasible to the site of infection. Different forms of nanoparticles (liposomes, lipid nanoparticles, and metal oxide nanoparticles) have direct applications in treating skin infections (Lin et al., 2021).

## 5 Nanocomposites and its varieties

**Figure 2:**
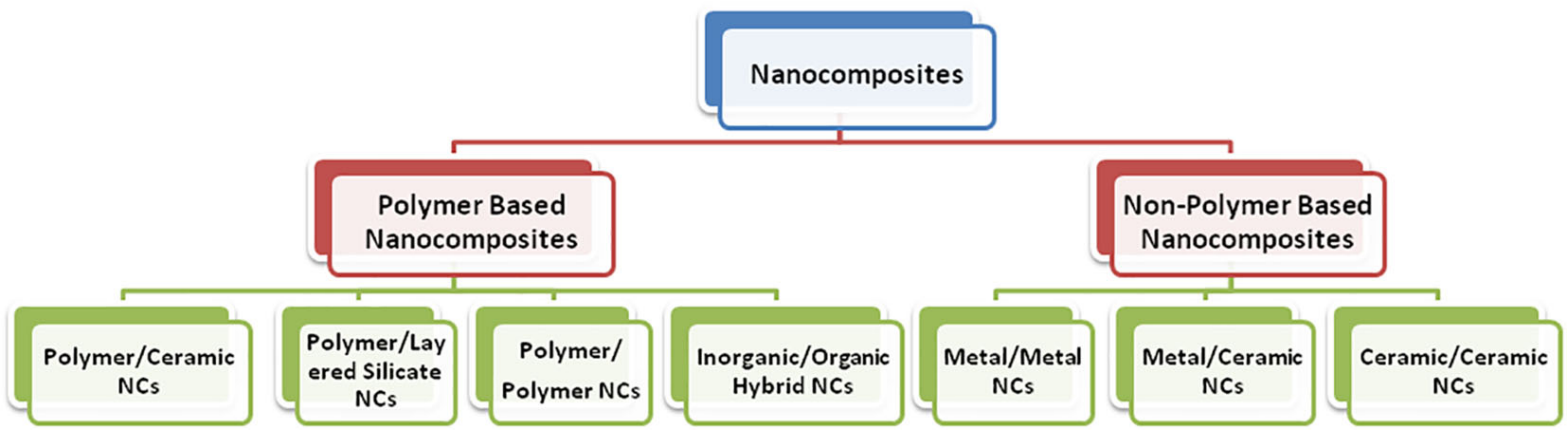
Types of nanocomposites.

The continuous demand of humans for more sophisticated technology for self-betterment brings forth a new class of materials where two different materials combine in such a way as to become a far different or an entirely new material. Such materials are called composite materials. In the present scenario, exploiting the exciting properties of nanomaterials at a very large scale is practically considered to design nanocomposites (Cho et al., 2014; Kandiah et al., 2015). The term “nanocomposites” was first coined by Komarneni, 1992; Harriott and Noverr, 2009). Nanocomposites are defined as a class of materials that contain at least one phase with constituents in the nanometer (1 nm = 10^–9^ m) domain. Additionally, as dimensions reach the nanometer level, interactions at phase interfaces become largely improved, and this is important to enhance the material’s properties. In this context, the surface area/volume ratio of reinforcement materials employed in the preparation of nanocomposites is crucial to the understanding of their structure–property relationships. The varieties of nanocomposites include polymer-based and non-polymer-based nanocomposites (**Figure 2**).

### 5.1 Polymer-based nanocomposites

Composites made up of polymer matrix and filler (clay, nanotubes, nanoparticles, etc.) with at least one dimension less than 100 nm are called polymer nanocomposites. Scratch-resistant transparent amorphous thermoplastic coating is the latest example of the modern application of polymer-based nanocomposite materials due to the small size of the fillers. Unlike traditional micron-filled composites, these novel fillers often alter the properties of the entire polymer matrix and, at the same time, impart new functionality because of their chemical composition and nanoscale size.

#### 5.1.1 Polymer/ceramic nanocomposites

When the single ceramic layer of nano range is homogeneously dispersed in a polymer matrix, it is called polymer/ceramic nanocomposites. Due to dipole–dipole interaction, ceramic layers tend to align parallel. An extensively used technique for polymer/ceramic nanocomposite is the sol-gel process. However, intercalation of a single polymer chain is the most suitable method to obtain ordered layered structures that can be acquired by polymerizing a monomer with a ceramic. A drastic change has been obtained in the properties of such composites due to a large number of polymer–ceramic interfaces at the nano-level and ordered polymer chains in the surroundings.

#### 5.1.2 Polymer/layered silicate nanocomposites

Polymer/layered silicate (PLS) nanocomposites are broadly classified as intercalated nanocomposites, flocculated nanocomposites, and exfoliated nanocomposites. Intercalated nanocomposites bear a regular fashion of polymer chain insertion into silicate layers with a distance of a few nanometers, regardless of the polymer-to-clay ratio. Here, the monomer is in a solution where silicate layers are swollen within. The polymerization is initiated between layers, expanding and dispersing the clays into the polymer (Kumar et al., 2009). An advantage of this method is the tethering effect, which allows nano-clays with a chemically active surface to link polymer chains during polymerization (Hussain et al., 2006). Flocculated nanocomposites consist of intercalated and stacked silicate layers flocculated (the process of clay aggregate into clot-like masses or precipitate into small lumps) to some extent due to the hydroxylated edge–edge interaction of the silicate layers. Exfoliated nanocomposites are composed of single silicate layers separated in the polymer matrix by average distances governed by the type of clay loading. To fabricate such nanocomposites, the process involves the use of solvent, in which polymer or prepolymer is soluble, to promote the exfoliation of layered silicate into single layers, and it is possible to break the weak forces that stack the layers together. Then, the polymer is easily absorbed onto the laminated sheets. After solvent evaporation, a sandwich of polymer is obtained by sheet reassembly (Alexandre and Dubois, 2000, Hussain et al., 2006). However, this process reveals commercial handling problems due to the solvent’s high costs and phase separation of the synthesized product from the solvent (Gao, 2004; 2013). Processing equipment and processing conditions are important parameters for clay dispersion. Along with this, polymer molecular weight also regulates clay dispersion. Polymers with a high molecular weight enhance clay dispersion.

#### 5.1.3 Polymer/polymer nanocomposites

It is assumed that appropriate scaling (near the nano range) of polymer can practically be achieved by employing block copolymers. The basic requirements for these nanocomposites are control over dispersion, morphology, and adhesion between immiscible phases. Strong mechanical stirring provides a good dispersion of polymer A in an immiscible polymer B. Block copolymers can be synthesized by various chemical methods opted as per requirement (Parhi et al., 2022; Sharwani et al., 2022).

#### 5.1.4 Inorganic/organic polymer nanocomposites

Metal clusters of nano range dispersed in a polymer matrix formed Metal/polymer nanocomposites. Owing different properties by nanoparticles are beneficial to incorporate them into a matrix material, e.g., a polymer solution or melt, or an elastomer. Zerovalent metal nanoparticles can readily be dispersed in an inert polymer thin film matrix. The ‘bottom-up’ approach to the synthesis of metal nanoparticles has largely relied on the colloidal route involving the reduction of metal ions by appropriate chemical reagents and stabilization by suitable surfactants. For thin film generation of composite metal/polymer, plasma-enhanced vapor deposition of polymer and simultaneous sputtering of any metal is well employed at low-temperature processing (Haghparasti and Shahri, 2018).

#### 5.1.5 Inorganic/organic hybrid nanocomposites

This group of nanocomposites bears both organic and inorganic components as constituents. Both homogeneous and heterogeneous systems are observed containing at least one component in the nano range. Again, the nature of the interface (based on the type of bonds, i.e., weak or strong) has been used to divide these materials into two distinct classes. In class I, both constituents are embedded and only weak bonds (hydrogen, van der Waals, or ionic bonds) give cohesion to the whole structure, while in class II materials, the constituents are linked together through strong chemical bonds (covalent or iono-covalent bonds). Indeed, the mild synthetic conditions offered by the sol-gel process (metallo-organic precursors, organic solvents, low processing temperatures, and processing versatility of the colloidal state) allow the mixing of inorganic and organic components at the nanometric scale. Due to the anti-corrosive nature of certain metal nanocomposites, there has been a profound application as an antibacterial coating in biomedical devices (Owhal et al., 2021). The stable mechanical and biological properties make hybrid nanocomposites beneficial in orthopedic implants. GC-induced implant failure and osseointegration failure can be cured with the aid of the organic–inorganic hybrid Lbl coating (Liu et al., 2013). This hybrid nanocomposite has a potential bactericidal role on MRSA and *E. coli*.

### 5.2 Non-polymer-based nanocomposites

Ceramics have good wear resistance and high thermal and chemical stability. However, they are brittle. To overcome this limitation, ceramic–matrix nanocomposites (metal/ceramic and ceramic/ceramic) have been receiving attention along with metal/metal nanocomposites.

#### 5.2.1 Metal/metal nanocomposites

Alloys were discovered and developed during the era of human civilization. New alloys continue to be sought after to meet future demands. Nanocrystalline alloys can be made using numerous techniques. However, numerous properties, including porosity, impurity, texture, and grain size distribution, are very essential and determined by the processing procedure. Kappa-carrageenan (CRG) synthesized by green synthesis has proved to be a potent antimicrobial agent for removing bacterial biofilms. CRG has direct application in the biomedical field and food packaging industry (Goel et al., 2019). In the case of alloy nanoparticles, the combination of gold and palladium is popular since these metals are miscible at any ratio. New qualities can be produced by simply combining two different metal nanoparticles together or using the sol-gel method. The main restriction, however, is an accumulation of components that reduces surface area. A few strategies, such as gas fluidization and the addition of an external force like vibration, sound waves, and magnetic excitation, are used.

#### 5.2.2 Metal/ceramic nanocomposites

Titanium-based nanocomposites are resistant to corrosion and have good biocompatibility. Dental implants have been using titanium with great success. Dental crowning is the prevalent implant, thereby protecting tooth from later damages. Ceramics, on the other hand, have improved strength. Calcium phosphate released near an implant improves bone apposition (Toussi et al., 2018). Metal/ceramic nanocomposite materials are used in high-temperature applications involving strength, stiffness, crack resistance, and other properties associated with structural materials in the aerospace industry. Carefully, agglomerate-free particles of the nano range will be synthesized *via* the sol-gel method. Partial hydrogen reduction of ceramic and metallic oxide mixture will be carried out at a specific temperature, yielding pre-sintering powder. Furthermore, optimization of processing parameters (temperature and pressure), which are mostly physical, is applied to obtain the desired product.

#### 5.2.3 Ceramic/ceramic nanocomposites

Biomaterials like artificial joint implants are one of the major thrust areas for ceramic nanocomposites. The ceramic-ceramic implants made of zirconia-toughened alumina nanocomposites have a potential lifespan of more than 30 years. Chemical methods include the sol-gel process, colloidal and precipitation approaches, and template synthesis. A large variety of parameters affecting the sol-gel process, such as type of solvent, timing, pH, precursor, and water/metal ratio, allow the versatile control of structural and chemical properties of the final oxide materials (Ennas et al., 1998).

### 5.3 Green nanocomposites

The use of chemicals have attracted attention from the public since they affect not just researchers and businesses but also the environment. To reduce this global concern, scientists have developed new ways to minimize it. In this context, natural products have played a vital role in fulfilling it. Mostly, plant parts and microbes are utilized for this purpose. Extracts of these have multiple roles, such as media for reactions, reducing agents, and capping agents. They are cheap as compared to chemicals, are easily available, and are easy to use—factors that contribute to the versatility of this technique, leading to the term “green.” Among various green nanoformulations, green nanocomposites have gained attention. To maintain a sustainable environment, green nanocomposites have played an important role through various applications in automotive, construction (Presting and Konig, 2003; Sharwani et al., 2022), packaging (Zare et al., 2019), and medical science (Bibi et al., 2021). They are based on eco-friendly materials. The biodegradable polymers generated provide solutions to the issues caused by synthetic and plastic waste. Green composites are composed of plant fibers and natural resins. We need to use synthetic polymers and/or additives as complements to green polymers. Green nanocomposites (either nanoscale natural macromolecules or biopolymers containing nanoparticles) are a class of scaffolds with acceptable biomedical properties (drug delivery and cardiac, nerve, bone, and cartilage as well as skin tissue engineering), enabling one to achieve the required level of skin regeneration and wound healing (Pathania et al., 2022).

## 6 Antimicrobial activity of nanocomposites against bacterial biofilms

Biofilm is a major problem in the field of medicine; it consists of a complex process that involves the attachment of bacteria to the surface, proliferation, extracellular polymeric substance (EPS) production, small colonies formation, and ultimately mature biofilm diffusion (Riau et al., 2019; Elshaarawy et al., 2020). Biofilm is a complex biological structure, consisting of a wide range of bacteria, which involves the association of an extracellular polymer matrix (EPM) with microorganism cultures. The bacteria respond to environmental cues that induce the synthesis of EPS. As a result, if the formation of EPS can be inhibited or prevented, then biofilm formation will also be restricted (Chávez de Paz et al., 2011; Riau et al., 2019). Bacterial biofilm formation is a major area of concern in the medical field as it can resist harsh conditions (Dokoshi et al., 2020) and is ineffective to traditional antibiotic therapies as compared to planktonic bacteria (Wu et al., 2015). Moreover, the majority of infections that are hard to treat are caused by biofilms, including urinary tract infections, catheter infections, and dental plaque formation (Lewis, 2001). Therefore, there is an urgent need to develop novel methods against antibacterial-resistant biofilm (Xu et al., 2021). However, nanoparticles are also not much effective against antibiotic resistance mechanisms (Wang et al., 2017). To overcome the resistance of biofilm against conventional antibiotic therapy, antimicrobial-loaded nanoparticles alone or combined with other substances could enhance the bactericidal activity of nanomaterials. This includes killing the pathogens effectively without harming other cells or causing any adverse effects on living cells (Thambirajoo et al., 2021). The use of nanotechnology in medicine is known as targeting. The goal of this strategy is to deliver medicines, diagnostics, including imaging agents, or both using nanoparticles (NPs) to target specific diseases and disorders. Targeting NP treatments are getting a lot of attention for identifying, treating, and managing neurodegenerative disorders. They have distinctive characteristics for targeting and for bridging the blood–brain barrier (BBB), which is well recognized. The characteristics of nanomaterials are different from conventional antimicrobial agents, which gives a new path for preventing and eradicating biofilm formation (Yuan et al., 2020). These nanocomposites are observed to have improved antimicrobial activity against resistant bacteria. Phytochemicals, which are antibiotics derived from plants, offer a viable alternative to conventional antibiotics. Their ineffectiveness in treating biofilm infections, however, is due to their limited solubility in aqueous media. Cross-linked polymer nanocomposite “sponges” were made with phytochemicals and incorporated for the treatment of bacterial biofilms. In addition, it was also demonstrated that nanocomposites with less hydrophobic phytochemicals have more potent antibiofilm efficacy (Li et al., 2019).

### 6.1 Nanocomposites targeting Gram-positive bacterial biofilm

There are various Gram-positive bacteria such as *S. aureu*s and *S. epidermidis* that impede the healing of wounds (Roy et al., 2020). Studies reported that nanocomposites are more effective than pure NPs; for example, CuO embedded with Zn nanocomposite showed increased antibacterial activity compared with pure CuO or ZnO (Malka et al., 2013). Moreover, TiO_2_-doped CuO and Li-doped MgO also have a significant antibacterial effect than pure MgO and TiO_2_ (Rao et al., 2013). Similarly, CeO_2_-CdO nanocomposites also had antimicrobial activity against Gram-negative bacteria (Magdalane et al., 2017). Furthermore, nanocomposite Mn and Fe ion-doped ZnO NPs have been observed to have higher antibacterial activity against a wide range of bacteria such as *S. aureus*, *Bacillus subtilis*, *Proteus mirabilis*, and *E. coli* compared with ZnO (Unal et al., 2018; Bahari et al., 2019). It was also seen that nanocomposites with metal oxides significantly improve the antimicrobial action through some external factor induction (Hsueh, 2010). Apart from this, a combination of three metal oxides like TiO_2_-ZnO-MgO and CuZnFe oxide nanomaterials have higher antibacterial activity in inhibiting biofilm synthesis (Alzahrani et al., 2018). CuZnFe oxide NPs were found to be more effective against *E. faecalis* than ZnO NPs but less effective than CuO (Alzahrani et al., 2018). Thus, the nanocomposite with the combination of three NPs affected the biofilm formation to a higher degree than alone (Alzahrani et al., 2018). The mechanism behind the metal oxide NPs is that oxide causes the slow release of metal ions, which disrupt cellular processes by penetrating the cell membrane (Hayden et al., 2012). Metallic NPs cause severe oxidative stress through the generation of reactive oxygen species (ROS). The ROS level is in turn correlated with the antibacterial activity due to its damaging effect to the bacterial cell membrane, disrupting the nucleic acids, inhibiting the enzyme, and thus obstructing the biofilm formation (Ali et al., 2019; Song et al., 2019). Some studies report the effects of different nanocomposites such as biodegradable crosslinked polymer-stabilized oil-in-water, effective against bacterial biofilm (Landis et al., 2018). Nanocomposites are observed to be effective against the antibiotic-resistant bacterial biofilm of Gram-positive bacteria. A study has reported that a nanocomposite with immobilized ciprofloxacin results in the reduction of biofilm formation in *S. aureus* of up to 85%. This nanocomposite has higher antibacterial activity against Gram-positive bacteria compared with Gram-negative bacteria (Rumyantceva et al., 2021). This showed that nanocomposites have better antibacterial properties and are effective in treating Gram-positive bacteria such as *S. aureus* (Xu et al., 2021). Furthermore, it was observed that the dendritic collagen matrix embedded dexamethasone-silver nanoparticle biofilm was formed, which involved *S. aureus* (Ramyaa Shri et al., 2021). Another study reported that using *Legistromia speciosa* floral extract, the synthesis of graphene oxide-silver nanocomposite (GO-Ag) inhibited the Gram-negative (*E. cloacae*) biofilm formation (Kulshrestha et al., 2017). Moreover, combining graphene quantum dots (GQDs) and OH– radicals was observed to inhibit the biofilm formation of *S. aureus* by damaging the intercellular components (Sun et al., 2014). Polymethyl methacrylate (PMMA) incorporation into CNTs could also reduce the infections caused by microbes, such as *S. mutans*, *S. aureus*, and *C. albicans*, by affecting microbial adherence (Kim et al., 2019). Nanocomposite GO with Ag impedes the growth of *S. aureus* by preventing cell mitosis of bacterial cells (Singh et al., 2018). These studies indicated that nanocomposites have excellent antibacterial ability against Gram-positive bacteria. Thus, it can be used as a novel antimicrobial agent for treatment.

### 6.2 Nanocomposites targeting Gram-negative bacterial biofilm

Nanocomposites are efficient against the development of resistant biofilms by Gram-negative bacteria as well as Gram-positive bacteria in various combinations. Same as Gram-positive bacteria, dendritic collagen matrix embedded dexamethasone-silver nanoparticles inhibit biofilm formation in the Gram-negative bacteria *K. pneumoniae* (Ramyaa Shri et al., 2021). A recent study has also observed that the AMP@PDA@AgNP nanocomposite has an inhibitory effect against Gram-negative bacteria, and *E. coli* and *P. aeruginosa* biofilm was higher than Gram-positive *S. aureus*. Furthermore, biofilm destruction in Gram-negative bacteria was also observed to be significantly different when compared with control (Xu et al., 2021). Moreover, nanocomposite immobilized with an antibiotic, ciprofloxacin, leads to reduced *E. coli* biofilm formation, and approximately 71% of its activity was even more effective compared with antibiotic alone (Rumyantceva et al., 2021). There are various studies that reported that, just like Gram-negative bacteria, there are various crosslinked polymer-stabilized oil-in-water and graphene oxide-silver nanocomposites that have antibacterial activity against Gram-negative bacteria biofilm formation (Kulshrestha et al., 2017; Landis et al., 2018). It has been seen that nanocomposites eradicated Gram-negative bacteria, including *P. aeruginosa*, *E. coli*, and *E. cloacae* complex in the biofilms (Li et al., 2019). These results indicated that nanocomposites are a better and novel approach for targeting resistant biofilm formation due to the wide range of Gram-negative bacteria. Apart from this, there were nanocomposites of copper, silver, or zinc NPs, embedded with zeolite, that were not effective against *V. cholera* biofilm formation, which showed that these bacteria have a resistance mechanism for these nanocomposites (Meza-Villezcas et al., 2019). Furthermore, the nanocomposite of silver NPs modified with curcumin causes oxidative stress through a hole in the membrane leading to antibacterial activity against *B. subtilis* and *E. coli* (Song et al., 2019). Similar to Gram-positive bacteria, metal oxides have antibacterial properties. For instance, mixing zinc and magnesium oxide with hydroxypropyl methylcellulose prevented *P. mirabilis* from forming a biofilm (Iribarnegaray et al., 2019). Another nanocomposite of octenyl succinic anhydride modified HA (OSA-HA) encapsulated antibiotic peptides, and poly D,L-lactic-co-glycolic acid (PLGA) disseminated the *P. aeruginosa* biofilm formation in lung mucus lining (Kłodzińska et al., 2019). Carbon NPs with an iron oxide nanocomposite have an antibacterial effect through the cell membrane, protein, and DNA degradation of *E. coli* (Alimardani et al., 2019). As we have already discussed, metal oxides have antibacterial activity, and ZnO, CuO, MgO, and TiO_2_ doped with other metal nanoparticles have higher antibacterial activity against Gram-negative such as *E. coli* than any of them in pure form (Malka et al., 2013; Rao et al., 2013). Furthermore, the combination of three metal oxide nanoparticles such as CuZnFe oxides reduces *E. coli* biofilm formation ability more than individual oxides of metal alone (Alzahrani et al., 2018). These oxides release metals slowly, which can penetrate the cell membrane and affect the cellular processes (Hayden et al., 2012). The toxic effect of ZnO can be minimized significantly by doping iron or Mg, which is safe for mammalian cells (Vidic et al., 2013; Ravichandran et al., 2015). In addition, nickel oxide also showed antibacterial activity against the *P. aeruginosa* biofilm. The mechanism behind the antibacterial activity of NiO is that it affects DNA replication and protein synthesis and alters the cell membrane, which also leads to cell death (Maruthupandy et al., 2020). A study reported that the ZnO/CdS nanocomposite impedes *P. aeruginosa* biofilm formation and also reduces the biofilm microbial population (Gholap et al., 2020). Moreover, the addition of Ag and Au in nanocomposites enhances their antibacterial potential against resistant *E. coli* (Matai et al., 2014); for example, Ag-SiO_2_ and Ag/Fe_3_O_4_ nanocomposites have increased antibacterial ability against *E. coli* (Mosselhy et al., 2017; Chang et al., 2018). The nanocomposite of ZnO NPs doped with Mn and Fe has also increased antibacterial activity against a wide range of bacteria such as *E. coli*, *K. pneumoniae*, *S. typhi*, *P. aeruginosa*, and *P. mirabilis* (Sharma et al., 2016; Bahari et al., 2019). Thus, as discussed previously, these nanocomposites with metal oxides have improved the efficacy of bacterial biofilm treatment through ROS generation and targeting bacterial DNA and proteins. Therefore, these results showed that choosing nanocomposites for biofilm treatment is the better way to treat biofilm-related infections.

## 7 Detection and characterization of bacterial biofilm

The World Health Organization (WHO) has previously raised concerns regarding bacterial biofilms in water pollution (Wingender and Flemming, 2011) and antibiotic resistance (Talebi et al., 2019), emphasizing the significance of developing novel and sensitive biofilm characterization strategies that can also be used to screen drugs that are specifically suited for biofilms. In this condition, innovative sensing techniques help deal with the biofilm problem using different approaches (Parlak and Richter-Dahlfors, 2020). Researchers have focused on the sensors and methods that identify the development of bacterial biofilms.

### 7.1 Sensors to investigate biofilm processes

The dynamics of certain indicators (such as oxygen level, pH, ions, and metabolite concentrations) within bacterial biofilms have been tracked using a variety of sensing techniques. The most effective method for a given application depends on several variables, including the type of target and the biofilm’s level of resistance to intrusive operations. For instance, highly sensitive and dependable electrochemical sensors are based on microelectrodes. To take the measurement, they must contact and pierce the biofilm, potentially endangering the biofilm’s structural integrity. Conversely, optical methods utilizing planar optodes and tiny particles are less intrusive. The biofilm’s oxygen and pH levels have been monitored using nanoparticles. Planar optodes, which are polymeric films embedded with oxygen-sensitive luminescent probes, and labeled micro- and nanoparticles have been employed in optical imaging systems based on confocal and fluorescence microscopy to monitor changes in oxygen and pH in the biofilm. Planar optodes, which are 2D polymer films on which the cells cling to form the biofilm, are unable to reveal information inside the biofilm. In contrast, micro- and nanoparticles doped with fluorescent or luminescent dyes, which are sensitive to changes in pH or oxygen levels in the biofilm, can be dispersed inside the EPS matrix, providing 3D mapping of the oxygen concentration and pH inside the biofilm. *In situ* studies of the biofilm can be conducted with less stress due to their non-destructive nature. The detection of heavy metal (Fe^3+^, Cu^2+^, Zn^2+^, and Hg^2+^) adsorption by bacterial biofilms that is pertinent to bioremediation applications has also been done using fluorescent probes (Funari and Shen, 2022).

The challenges of detecting biofilm in complex food samples, such as the characterization of biofilm formation mechanisms, identification of microbial metabolic activities, diagnosis of potential food pathogens, and sanitation monitoring of food processing equipment, have been taken on by optical nanosensors (Pu et al., 2021). Recently, nanosensors have been developed for biofilm optical detection with high sensitivity and great spatial resolution at nanoscale scopes that can convert biological information into optical signals (Talebi et al., 2019). Nanosensors, which are usually referred to as nanoprobes and nanozymes in microbiological detection due to their varied optical properties, can be tuned and designed for bacterium sensing and chemical recognition. The primary focus of nanosensors is on their capacity to transform chemical data from biomolecules in a microscopic environment into signals appropriate for detection. As a result, fluorescent, SERS, and colorimetric nanosensors make up the majority of optical nanosensors (Talebi et al., 2019).

Funari et al. have demonstrated that the utilization of localized surface plasmon resonance (LSPR) is done for label-free, *in situ*, real-time monitoring of *E. coli* biofilm formation on a gold mushroom-shaped nanoplasmonic substrate. By tracking the wavelength change in the LSPR resonance peak with fine temporal precision, the LSPR sensor can detect the traces of biofilm growth in real time (Funari et al., 2018). This sensor functions to analyze how different medications, such as kanamycin and ampicillin, as well as rifapentine, affect biofilm formation, preventing cell adhesion (Funari et al., 2018). LSPR-based technology is flexible and easy to use, making it a great tool for biofilm characterization and drug screening. It can be applied to a wide range of therapeutically relevant bacteria and provides an invaluable tool for drug screening and biofilm characterization (Funari et al., 2018).

There are recent advancements in biofilm prevention and control by using active antibiofilm nanocoatings. Antibiofilm tactics based on antimicrobial substances that eliminate infections and prevent their growth or interfere with the molecular pathways underlying the biofilm-associated rise in resistance and tolerance have been developed (Balaure and Grumezescu, 2020). These substances, which come in a variety of chemical forms, work in a variety of ways to target important bacterial metabolic pathways, cellular components including cell walls and membranes, or processes that underlie various stages of the biofilm life cycle. Eventually, smart antibiofilm coatings have been introduced that release their antimicrobial payload when necessary and are activated by a variety of triggers, including changes in the environment’s pH or temperature or enzymatic triggers.

Oxygen-sensitive luminescent nanosensors that can be incorporated into biofilms to study oxygen penetration, distribution, and antibiotic efficacy were successfully tested (Jewell et al., 2019). Sensors were used to track how antibiotics affect metabolism in biofilms made from clinical isolates. It not only provides a non-disruptive method for imaging and detecting oxygen in biofilms but also shows how to adapt a nanoparticle-based sensing platform to measure a variety of ions and small-molecule analytes (Jewell et al., 2019).

### 7.2 Quorum sensing disruption/molecular pathway inhibition of quorum sensing molecules

Antibiotic resistance among bacteria has evolved because of profound antibiotic stress. Likewise, *P. aeruginosa* evolve an elevated level of resistance to antibacterial agents *via* quorum sensing (QS) (Sadoq et al., 2023). With the advancement of antiviral therapies, the bacterial infection can be constrained without offending its resistance. In the phenomenon called QS, the bacteria correspond to each other in a density-dependent manner. This communication between the bacteria can be interrupted by disarming quorum-sensing systems. Such a strategy would save an ample amount of investment in the pharmaceutical industry. The Rhll/R system of *P. aeruginosa* is involved in the formation of biofilm. Molecular docking studies have shown the capability of silver, zinc, and copper oxide-based interactions with the acyl-homoserine-lactone synthases (LasI and RhII), thereby impeding the synthesis of functional signaling molecules. There are some regulatory proteins (LasR and RhIR) as well, which can bind to the nanoparticles, reducing the expression of QS-controlled genes (Sadoq et al., 2023).

These nanoparticles thwart the process of biofilm formation. Biofilm-based infections can be cured by intruding on the QS signaling cascade. pH, signal flow rate, and nutrient availability regulate QS-mediated biofilm function (Mishra et al., 2023). QS works by binding signaling molecules to the microbial receptors, instigating the activation of the gene network. Histidine kinase receptors sense the QS signals regulating gene expression in Gram-positive bacteria, which is nothing but the two-component regulating system, whereas receptive cell regulatory proteins after attaching to AHLs regulate the gene network in Gram-negative bacteria (Kalia, 2013).

Microbiology, nanotechnology, and optical technology are all involved in the multidisciplinary field of study on the imaging, identification, and chemical characterization of biofilms using optical nanosensors. Future studies on biofilm control will have a theoretical foundation due to the data collected by optical nanosensors, which include information on changes in biochemical contents as well as their spatial distribution and morphology.

## 8 Potential combating strategies

**Figure 3:**
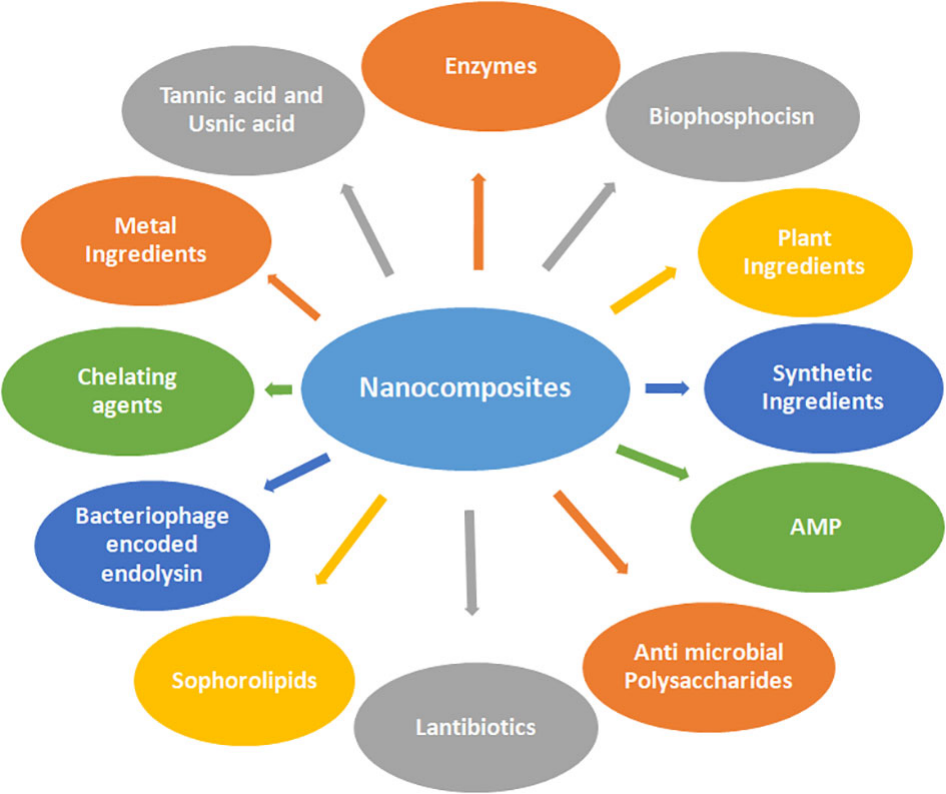
Various anti-biofilm molecules tested so far include herbal active compounds, chelating agents, peptide antibiotics, antibiotics, and synthetic chemical compounds involving nanocomposites.

Microbial evolution has led to bacterial biofilms gaining resistance to broad-spectrum antibiotics, which is the main cause of morbidity and mortality. To overcome the challenge of these drug-resistant superbugs, several antimicrobial nanocomposites act as weapons (Roy et al., 2018). Various anti-biofilm molecules tested so far may include herbal active compounds, chelating agents, peptide antibiotics, antibiotics, and synthetic chemical compounds as summarized in **Figure 3**.

### 8.1 Enzymes

They are a good choice to attack the unique target sites either in bacterial cell walls or non-selectively by generating bactericidal molecules. The antimicrobial properties of enzymes are attributed to their tendencies to aggregate or incorporate polymeric nanocomposites, which have potential in human therapeutics (Wu et al, 2017). Dispersin B (glycoside hydrolase), along with cefamandole nafate, is found to be a highly promising tool for preventing bacterial colonization of medical devices against *S. epidermidis*. Deoxyribonuclease I is also a potent anti-biofilm agent that acts by degrading extracellular DNA present in the matrix and rendering the matrix weaker and more susceptible to antimicrobials. DNase acts as a better anti-biofilm agent in individual biofilm formation than against the mixed species biofilms (Donelli et al., 2007; Sharma and Singh, 2018).

### 8.2 Biophosphocins

They are also known as nubiotics, which act as novel antimicrobials for enhanced killing of antibiotic-resistant and biofilm-forming bacteria. These are small protonated deoxynucleotide molecules that cause depolarization of bacterial cell membrane and also penetration of bacterial biofilms leading to death of encased bacteria (Wong et al., 2015).

### 8.3 Plant ingredients

The most abundant polyphenol found in green tea (*Camella sinesis*) is epigallocatechin gallate (EGCG) (Vidigal et al., 2014). Ellagic acid, esculetin, and fisetin are natural ingredients of dietary plants where fisetin was found to inhibit biofilm formation by *S. aureus* and *S. dysgalactiae* in a better manner than esculetin and ellagic acid. Esculetin, in turn, is found to be more efficient in preventing biofilms by *S. aureus* than ellagic acid. Ellagic acid prevents biofilm formation by *S. dysgalactiae*. Ellagic acid is also obtained from green tea while sources for esculetin are *Santolina oblongifolia*, *Tagetes lucida*, and Alchemilla speciosa, and those for fisetin are *Vitis vinifera, Cucumis sativa, Fragaria ananassa, Allium cepa, Solanum lycopersicum,* and *Malus domestica* (Durig et al., 2020).

Chitosan (a polysaccharide), curcumin (a beta diketone), eugenol (a phenol), and linoleic acid (omega-6-fatty acid) are antibacterial while berberine and resperine suppress biofilm formation in *K. pneumoniae*. The source of curcumin is Curcuma longa; that of eugenol is Syzigium aromaticum and Ocimum plants; that of linoleic acid is Coptis chinensis, Berberis vulgaris, B. aquifolium, B. aristata, and Hydrastis canadensis; that of berberine is Berberis aristata, B. vulgaris, and B. aquifolium; and that of Resperine is Rauwolfia serpentine and R. vomitoria (Magesh et al., 2013). Quercetin is obtained from Usnea longissima and it acts against biofilms of K. pneumoniae, Campylobacter violaccum, and P. aeruginosa (Gopu et al., 2015).

### 8.4 Synthetic ingredients

The synthetic halogenated Furanone (F-56) compound is derived from natural Furanone. It is produced by Australian macro alga *Delisea pulchra* and causes detachment of biomass of *E. coli* and *P. aeruginosa* (Hentzer et al., 2002).

### 8.5 Antimicrobial peptides

A previous study demonstrated the effect of an antimicrobial peptide derived from the stomach of the toad, Buforin-II, which enters the cell and binds to the nucleic acid to kill it without any membrane permeabilization (Cho et al., 2009). A small lytic peptide PTP-7 was found to be capable of diffusing into deeper layers of *S. aureus* biofilms to kill the bacteria effectively, thus providing a very good alternative to antibiotics for controlling infections related to bacterial biofilms (Kharidia and Liang, 2011). Two antibacterial peptides, cecropin P1 and PR-39, can be obtained from the intestine of a pig. Cecropin can kill *E. coli* in association with more peptides while PR-39 is capable of killing wild-type *E. coli* (Boman et al., 1993). Indolicidin was isolated from bovine neutrophils and found to inhibit *E. coli* DNA synthesis, making this antimicrobial agent of significant use in human therapeutics (Subbalakshmi and Sitaram, 1998). The antibiotic microcin B17 is a bactericidal peptide that acts as a poison for the DNA replication enzyme DNA gyrase, hence killing the bacteria (Vizan et al., 1991). Similarly, lytic peptide (PTP-7), a synthetic analog from Gaegurin 5; LL-37, a human cationic host defense peptide; PMAP-23, a cathelicidin-derived peptide identified from porcine leukocytes; and sushi peptides, derived from the sushi-3 domain of factor C, which is an LPS-sensitive serine protease of horseshow crab coagulation cascade, are potent anti-biofilm agents. Peptide 1018 is a small peptide that has the potential to eradicate *Acinetobacter baumannii*, *E. coli*, *K. pneumoniae, P. aeruginosa*, *S. aureus*, and *Salmonella typhimurium* biofilms at high doses and even checks the formation of biofilms (Fuente-Núñez et al., 2014). This peptide is derived from bovine host defense peptide bactenecins found in neutrophil granules.

### 8.6 Antimicrobial polysaccharides

CFT073 group II capsular polysaccharide is produced by extraintestinal *E. coli* of phylogenetic group B2 or D. These have the potential to inhibit biofilm formation by *E. faecalis, E. coli, K. pneumoniae, P. aeruginosa, S. aureus*, and *S. epidermidis* (Rendueles et al., 2013). Two additional anti-biofilm polysaccharides, Pel and Psl, were identified in a similar study and were found to be effective against *E. coli* and *Staphylococcus epidermidis* biofilms (Qin et al., 2009; Liang, 2015).

### 8.7 Lantibiotics

Gallidermin, which is obtained from *Staphylococcus gallinarum*, is found to prevent biofilm formation by *S. aureus* and *S. epidermidis* (Saising et al., 2012). Subtilin is another antibiotic that is considered similar to Nisin. Both are known to destroy bacterial biofilms by creating membrane pores in *S. simulans* and *B. subtilis* cells. Subtilin is sourced from *B. subtilis* Strain ATCC6633, Nisin is sourced from *Lactococcus lactis*, and Epidermin is sourced from *S. epidermidis* (Parisot et al., 2008).

### 8.8 Sophorolipids

These are the category of hydrophobic or hydrophilic biosurfactants that are produced on microbial cells or excreted extracellularly. Their antimicrobial property is attributed to the tendency to disrupt biofilms and the prevention of biofilm formation (Rienzo et al., 2015). Colistin is a polymixin E, obtained from *Paenibacillus polymyxa*, and is known to check biofilm formation against *S. aureus* for cystic fibrosis infections in lungs (Vidigal et al., 2014), while polymixin B is another biosurfactant and has the potential to combat multidrug-resistant bacteria and their biofilms (Park et al., 2011).

### 8.9 Chelating agents

An interesting study reported that chelating agents can disrupt the architecture of biofilms to destabilize bacterial membranes (Shadia and Aeron, 2014). Sodium citrate, tetrasodium EDTA, and disodium EDTA with tigecycline or gentamicin could reduce biofilms of *Staphylococcus* species and *P. aeruginosa.*

### 8.10 Bacteriophage-encoded endolysin

A bacteriophage-encoded enzyme endolysin, Ply C, can potentially act against the biofilms of *Streptococcus* (Shen et al., 2013). Ply C acts as a cell wall hydrolase enzyme to destroy the static and dynamic biofilms by diffusing through the matrix. It interacts with the cell surface carbohydrate that is peptidoglycan of the cell wall and has one enzyme active domain (EAD) to hydrolyze peptidoglycan bonds and another cell binding domain (CBD) in it (King et al., 2021).

### 8.11 Metal ingredients

Silver and iodine are employed as antimicrobials to help suppress the biofilms in chronic wounds (Percival et al., 2014). Octenidine hydrochloride, chlorhexidine, and cadexomer iodine polyhexamethylenebiguanide are also known antimicrobial agents. Gold nanoparticles integrated with graphitic carbon nitride (g-C3N4) have bactericidal effects against drug-resistant *E. coli* and *S. aureus* when combined with hydrogen peroxide (Wang et al., 2017). This has not only reduced bacterial infections but also accelerated wound healing rate.

### 8.12 Tannic acid and usnic acid

Tannic acid was found to inhibit surface colonization in *S. aureus* (Payne et al, 2013) while usnic acid is a natural secondary lichen metabolite that acts as an antimicrobial agent for inhibiting the formation of bacterial biofilms even on polymeric surfaces (Francolini et al, 2004) and also inhibits the virulent morphological traits of *Candida albicans* (Nithyanand et al, 2015).

## 9 Advantages of nanocomposites

One advantage of nanocomposites is their antimicrobial activity against Gram-negative planktonic bacteria and Gram-negative bacterial biofilms. These nanocomposites are capable of eradicating 90% of bacteria in biofilms and preventing infectious bacterial diseases. Nano-carriers show great anti-biofilm potential due to their small size and strong permeability. Nanocomposites can act on the formation and the diffusion stage of biofilms. They target biofilms, destroy their extracellular polymeric substances, and enhance biofilm permeability for antimicrobial substances. They improve the antibacterial ability of antibacterial drugs in the biofilm. This is acquired by protecting the loaded drug and controlling the release of antimicrobial substances. Therefore, polymer nanocomposites show superior mechanical properties, structural and thermal stability, promising electrical conductivity, and noise damping. They are corrosion-resistant and allow low permeability of fluids. They have lower density as compared to ceramic or metallic materials. They have low filter content and are easy to manufacture.

Epigallactocatechin gallate (EGCG) in patients with cystic fibrosis not only inhibited the formation of biofilms of *Stenotrophomonas maltophilia* but also reduced the number of viable cells in young and mature biofilms, thereby displaying promising therapeutic properties against *S. maltophilia* infection in cystic fibrosis (Vidigal et al., 2014). It shows a reduction in cell viability in mature biofilms, exhibiting its bactericidal properties. Ellagic acid, esculetin, and fisetin can be used to form antimicrobial drugs against a large number of infections. Chitosan, curcumin, eugenol, linoleic acid, berberine, and resperine-based nanocomposite drugs can fight against respiratory tract infections, urinary tract infections, and septicemia, especially in immunocompromised individuals. This is carried out by controlling and suppressing biofilm formation in *K. pneumoniae* (Magesh et al., 2013). Quercetin serves as a novel QS-based antimicrobial drug to manage food-borne pathogens (Gopu et al., 2015).

Synthetic halogenated furanone (F56) compounds can be used for the development of novel antibiotic, antimicrobial, or antipathogenic agents that hinder cell-to-cell communication in bacteria, making them less virulent and more sensitive to such therapies (Hentzer et al., 2002). These nano enzymes are more stable and cheaper than natural enzymes. Due to their small size, cells can easily take them up, and penetration into tissues and diffusion through the extracellular matrix allow them to reach atherosclerotic plaques, tumors, and biofilms (Benoit and Koo, 2016). The multifunctional nanocomposites can be exploited for their innovative biomedical applications to treat biofilm-related infections and also prevent oxidative stress. A nanocomposite formed from a V**_2_** O**_5_** nanowire (glutathione peroxidase) and MnO**_2_** nanoparticle [superoxide dismutase (SOD) and catalase] was found to be effective against removing ROS in a rodent inflammation model (Huang et al., 2016). These catalytic nanomaterials having enzyme-like properties lead to the disruption of biofilms, hence preventing neurodegeneration, tumor prevention, and wound healing. Ceria-based nanocomposites can mimic SOD and have anti-inflammatory and neuroprotective activities (Hirst et al., 2009) besides protecting cardiac progenitor cells and preventing retinal degeneration (Chen et al., 2006), in-stent coatings (Son et al., 2015), and reducing injury and ischemia in stroke (Kim et al., 2021).

## 10 Toxicity of nanocomposites

Nanotoxicology is a specialized branch that addresses the issues related to the toxicity of nanoparticles, nanocomposites, and other nanomaterials. It deals with the study of the adverse effects of engineered nanomaterials or nanoparticles on living organisms. The fragment of nanocomposites is found during their synthesis, application, and disposal. The workers associated with nanocomposite cutting, drilling, and sanding of nanocomposites have a high risk of developing occupational diseases. Nanocomposite fragments in the nano- and microscale are released due to reasons such as photodegradation, incineration, mechanical stress, thermal stress, and interaction with the liquids (Pacheco and Buzea, 2021). The airborne nature of the nanoparticles and nanofibers shows potential toxicity due to inhalation. It has been revealed that the main toxic effects induced by TiO_2_ are triggered by oxidative stress, which leads to cell necrosis and DNA damage (Han et al., 2020). The size and physical properties of NPs are partly responsible for NP toxicity, with particles with smaller sizes and higher stability being more interactive with lung cells (Bengalli et al., 2019). Although iron oxide nanoparticles are used in biomedical applications, numerous reports have shown that they also exhibit toxicity to normal healthy cells. Similarly, the cytotoxicity of superparamagnetic iron oxide nanoparticles (SPIONs) to actin cytoskeleton modulation, gene expression profile alteration, iron homeostasis disturbance, impaired alterations in signaling pathways, cell regulation, DNA damage, and oxidative stress has been demonstrated (Singh et al., 2010). A literature survey also reported that the combination of silver and iron oxide nanoparticles as nanocomposites holds exclusive and unique antimicrobial activities (Trang et al., 2017). Even though iron oxide nanoparticles are used in biomedical applications, numerous reports showed that they also exhibit toxicity towards normal healthy cells. To create safe nanocomposites, it is important to determine the types of nanomaterials that pose a health risk and to limit their use or mitigate their toxicity.

## 11 Challenges of antibiotic resistance biofilm

Antibacterial resistance presents difficulties for modern medicine because the spread and emergence of resistant bacteria have reduced the efficiency of medicines. Bacteria can resist antibiotic effects in several ways, such as altering the antibiotic’s target site, destroying or altering the antibiotic, efflux of antibiotics through efflux transporters, and reducing the influx of antibiotics by reducing membrane permeability (Munita and Arias, 2016). A substantial number of antibiotics on the market right now are useless because they lack the necessary minimum bactericidal concentration (MBC) and minimum inhibitory concentration (MIC), making them ineffective against infections. The disruption of desired bacterial biofilms’ EPS matrix is accomplished through newer, or matrix-centered, tactics, such as controlling microbial metabolism, matrix-degrading enzyme, photodynamic therapy, naturally occurring chemicals, QS, and nanotechnology.

Since they can be used as an alternative to treat infections caused by biofilm and multidrug resistance, nanoparticles are of utmost importance (Pelgrift and Friedman, 2013). Their nano-formulations can overcome many of the limits and limitations of conventional therapy. These formulations can pass through biological barriers. In the past, several nanoparticles, including metal nanoparticles, green nanoparticles, and many other combinations, have been utilized as antibacterial and anti-biofilm agents (Khan et al., 2021). Numerous studies on the eradication of bacterial biofilm communities based on nanoparticles have been published (Kulshrestha et al., 2014). Nanoparticles with strong antibacterial capabilities, such as copper, oxide, silver, zinc, and quantum dots, have been shown to have potential in the fight against biofilms (Zaidi et al., 2017). By raising oxidative stress, promoting cytoplasmic leakage, and denaturing the metabolic proteins, nanoparticles carrying ROS harm bacteria’s cell membranes and cell walls (Shoji and Chen, 2020). It causes changed cellular functioning and has an impact on bacterial cells’ physiological processes (Zaidi et al., 2017). Numerous *in vivo* experiments showed that nanoparticles can be utilized to combat a wide range of Gram-positive and Gram-negative bacterial strains with little cell toxicity and great compatibility (Li et al., 2016; Qayyum and Khan, 2016). It results in the potential use of nanoparticles as a treatment for bacterial infections (Khan et al., 2020). Kulshrestha and his coworkers reported CaF2-NP’s suppressive effect on genes associated with virulence factors, such as vicR, ftf, gtfC, come, and spam of *S. mutans*, and demonstrated enzymatic activity inhibition associated with cell adhesion, glucan synthesis, acid production, QS, and acid tolerance (Kulshrestha et al., 2016). The capacity of currently available antibiotics to treat infections that are currently resistant to accessible current therapies may be improved by inhibiting biofilm resistance. However, more research is required to completely understand the mechanism of antibiotic resistance in biofilms. As discussed above, keeping in mind the toxicity effects of nanocomposites, a strict balance has to be maintained while using nanocomposites to create new therapeutic approaches.

## 12 Conclusion

Biofilms are organized and complex communities in which bacteria communicate with each other and gain tremendous advantages. They spread over the outer and inner parts of the entire human body including organs and bones. Few to hundreds of bacterial species are associated with this phenomenon. Traditional antibiotics have covered a large period in medical history, but the ultimate solution to this issue remains unanswered. Moving forward, through the application of nubiotics, enzymes, natural products, antimicrobial peptide, etc., along with various formulations, novel pathways toward treatment have been created but still had limitations. In the present era of nanotechnology, many promising nanomaterials have been suggested as potential candidates. These nanocomposites are a new emerging class of compounds of biomaterials that have a hybrid property of participating ingredient molecules/groups. This feature is unique and can be harvested for this problem as it has an open arena to manipulate the toxicological and other relevant issues related to the candidate molecule. It is thus suggested that with this tool for the treatment of biofilm infection, more sensitive methods *via* a synergistic approach can be designed.

## Author contributions

AV, AW, and RS designed, conceived, wrote, and edited the manuscript. AV, ND, and RS prepared the figures. ND, NP, VU, and AM helped in writing and editing. AV, AW, and RS critically reviewed the manuscript. All authors contributed to the article and approved the submitted version.
